# Novel Galectins Purified from the Sponge *Chondrilla australiensis*: Unique Structural Features and Cytotoxic Effects on Colorectal Cancer Cells Mediated by TF-Antigen Binding

**DOI:** 10.3390/md22090400

**Published:** 2024-08-31

**Authors:** Ryuhei Hayashi, Kenichi Kamata, Marco Gerdol, Yuki Fujii, Takashi Hayashi, Yuto Onoda, Nanae Kobayashi, Satoshi Furushima, Ryuya Ishiwata, Mayuka Ohkawa, Naoko Masuda, Yuka Niimi, Masao Yamada, Daisuke Adachi, Sarkar M. A. Kawsar, Sultana Rajia, Imtiaj Hasan, Somrita Padma, Bishnu Pada Chatterjee, Yuji Ise, Riku Chida, Kayo Hasehira, Nobumitsu Miyanishi, Tatsuya Kawasaki, Yukiko Ogawa, Hideaki Fujita, Alberto Pallavicini, Yasuhiro Ozeki

**Affiliations:** 1 Graduate School of NanoBio Sciences, Yokohama City University, 22-2, Seto, Kanazawa-Ku, Yokohama 236-0027, Japan; 2 Department of Chemistry, KU Leuven, 3001 Heverlee, Belgium; w175502f@yokohama-cu.ac.jp; 3 Graduate School of Biomedical Sciences, Yokohama City University, 1-7-29 Suehiro-cho, Tsurumi-ku, Yokohama 230-0045, Japan; 4 Department of Life Sciences, University of Trieste, Via Licio Giorgieri 5, 34127 Trieste, Italy; pallavic@units.it; 5 Graduate School of Pharmaceutical Sciences, Nagasaki International University, 2825-7 Huis Ten Bosch, Sasebo 859-3298, Japan; kawasakit@niu.ac.jp (T.K.); yogawa@niu.ac.jp (Y.O.); fujita@niu.ac.jp (H.F.); 6 School of Medicine, Yokohama City University, 3-9 Fukuura, Kanazawa-Ku, Yokohama 236-0004, Japan; 7 emukk LLC, Kuwana 511-0902, Japan; 8 Department of Chemistry, Faculty of Science, University of Chittagong, Chittagong 4331, Bangladesh; akawsarabe@yahoo.com; 9 Center for Interdisciplinary Research, Varendra University, Rajshahi 6204, Bangladesh; rajia_bio@yahoo.com; 10 Department of Microbiology, Faculty of Biological Science, University of Rajshahi, Rajshahi 6205, Bangladesh; hasanimtiaj@yahoo.co.uk; 11 Department of Biochemistry and Molecular Biology, Faculty of Science, University of Rajshahi, Rajshahi 6205, Bangladesh; 12 Department of Oncogene Regulation, Chittaranjan National Cancer Institute, Kolkata 700026, Indiacbishnup@gmail.com (B.P.C.); 13 Kuroshio Biological Research Foundation, 560 Nishidomar, Otsuki, Hata, Kochi 788-0333, Japan; libertador0429@gmail.com; 14 Graduate School of Food and Nutritional Sciences, Toyo University, 48-1, Oka, Asaka, Saitama 351-8510, Japan; s3c102300053@toyo.jp (R.C.); suzuki284@toyo.jp (K.H.); miyanishi@toyo.jp (N.M.)

**Keywords:** *Chondrilla australiensis*, galectin, Porifera, TF-antigen, signal peptide, cytotoxicity

## Abstract

We here report the purification of a novel member of the galectin family, the β-galactoside-binding lectin hRTL, from the marine sponge *Chondrilla australiensis*. The hRTL lectin is a tetrameric proto-type galectin with a subunit molecular weight of 15.5 kDa, consisting of 141 amino acids and sharing 92% primary sequence identity with the galectin CCL from the congeneric species *C. caribensis*. Transcriptome analysis allowed for the identification of additional sequences belonging to the same family, bringing the total number of hRTLs to six. Unlike most other galectins, hRTLs display a 23 amino acid-long signal peptide that, according to Erdman degradation, is post-translationally cleaved, leaving an N-terminal end devoid of acetylated modifications, unlike most other galectins. Moreover, two hRTLs display an internal insertion, which determines the presence of an unusual loop region that may have important functional implications. The characterization of the glycan-binding properties of hRTL revealed that it had high affinity towards TF-antigen, sialyl TF, and type-1 N-acetyl lactosamine with a Galβ1-3 structure. When administered to DLD-1 cells, a colorectal carcinoma cell line expressing mucin-associated TF-antigen, hRTL could induce glycan-dependent cytotoxicity.

## 1. Introduction

Lectins, which are representative glycan-binding proteins, exhibit a diverse range of structural families and glycan-binding properties across various organisms [1]. They are potentially involved in a plethora of biological roles, which include defense, stress response, cell adhesion, and growth regulation [2,3,4]. According to the UniLectin structural database [5], approximately one-quarter of the lectins discovered to date bind to galactose, indicating that many non-reducing terminal galactoses in glycans are targets for lectin binding.

Sponges (phylum Porifera), the most early-branching group of animals, first appeared during the Ediacaran period, in the Neoproterozoic era [6]. They are sessile invertebrates living in a broad range of aquatic environments, from intertidal coastal waters to the deep sea, with about 5000 described species [7]. Consistently with their ancient origins deeply rooted in the stem of animal phylogeny, sponges possess lectins belonging to different families, such as galectins [8,9,10,11,12,13,14,15,16,17], C-type lectin [18], bacterial fucose-binding lectin [19], and tachylectins [20].

The genus *Chondrilla* (order Chondrosida) includes more than 16 extant marine species, and its members are characterized by collagen-rich tissues [21]. Different types of lectins have been previously discovered in several species of this genus. For example, a homotetrameric galectin has been purified from *Chondrilla caribensis*, a species that inhabits the Gulf of Mexico and the South Atlantic Ocean. This galectin exhibits antimicrobial, antibiofilm, and leishmanicidal activities [10,11].

In this study, we report the purification of a novel galectin from a different species of *Chondrilla* living in the Pacific Ocean, i.e., *Chondrilla australiensis*. Although hRTL was evolutionarily related to other galectins, it displayed some peculiar features, which most notably included an N-terminal extension, according to combined N-terminal sequencing by the Edman degradation and the analysis of RNA sequencing data. It likely serves as a signal peptide for secretion. Furthermore, hRTL exclusively displayed binding affinity towards the Thomsen–Friedenreich (TF)-antigen (Galβ1-3GalNAc) [22,23] and type-1 LacNAc (Galβ1-3GlcNAc). Notably, the TF-antigen is a characteristic oncogenic oligosaccharide in digestive carcinoma cells [24,25]. We show that administering hRTL to cells expressing TF-antigens induces cell death, accompanied by the activation of MAP kinases related to apoptosis.

## 2. Results

### 2.1. Purification of Chondrilla australiensis Lectin (hRTL)

**Figure 1:**
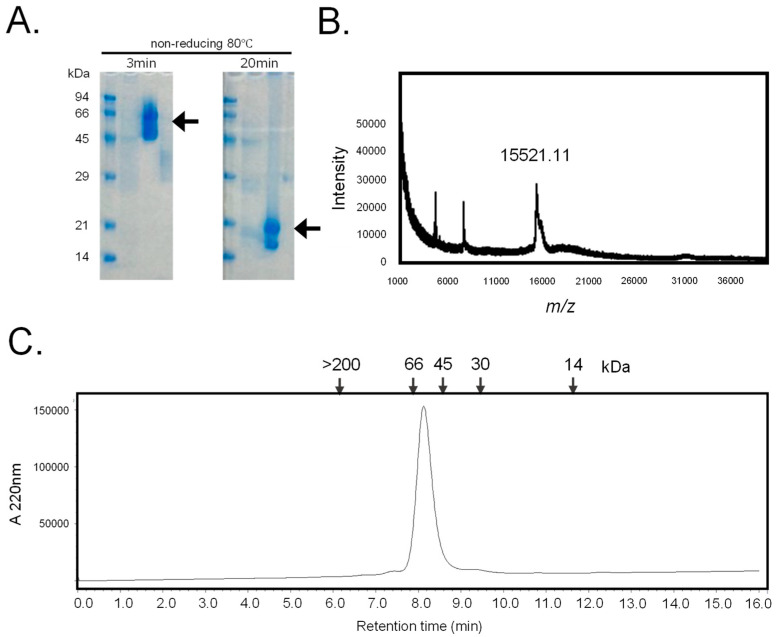
Purification of hRTL. (**A**) The SDS-PAGE pattern obtained after 3 min and 20 min of heating under non-reducing conditions. Numbers on the left indicate the molecular mass (kDa) of marker proteins. Arrows mark the positions of the lectins. (**B**) Mass spectrometry of hRTL. The MALDI–TOF-MS spectrum showed a molecular mass of 15,521.11 Da. One hundred laser shots measured the signal. (**C**) The gel permeation chromatography pattern. The purified lectin was applied on a Shodex KW 402.5-4F column connected to an HPLC pump at a flow rate of 0.33 mL/min. Numbers in the upper part indicate the following standard molecular markers: blue dextran (>200 kDa); bovine serum albumin (66 kDa); ovalbumin (45 kDa); carbonic anhydrase (30 kDa); horse myoglobin (14 kDa). The absorbance was detected at 220 nm.

The crude extract from *C. australiensis* lectin was applied to a lactosyl-agarose column. After washing the column with 50 mM sodium bicarbonate-containing saline, the desired lectin was eluted using a 50 mM lactose-containing buffer. SDS-PAGE analysis revealed that the lectin had a molecular mass of 66 kDa under non-reducing conditions and of 18 kDa under heated and short-heated conditions, respectively (Figure 1A). The molecular mass of the polypeptide chain was determined by mass spectrometry to be 15,521.11 Da (Figure 1B). Analysis by high-performance liquid chromatography coupled with a gel permeation column indicated a molecular mass of 60 kDa (Figure 1C). These results indicate that the β-galactoside-binding lectin from the sponge *C. australiensis* has a tetrameric structure, with non-covalently bonded subunits of 15 kDa. This molecule was named hRTL (*Chondrilla australiensis* tetramer lectin).

**Figure 2:**
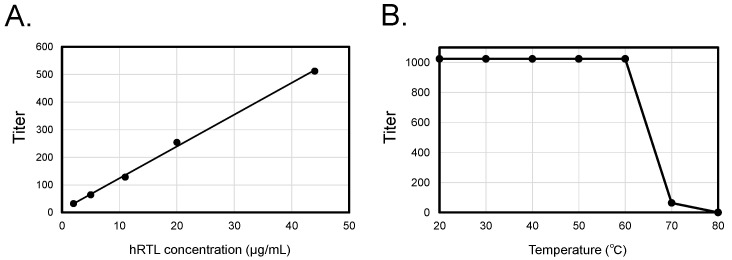
Concentration-dependent enhancement and thermal stability of hRTL hemagglutination activity. Co-relationship between concentrations, hemagglutination (**A**), and thermotolerance (**B**) of hRTL.

12.8. mg of hRTL was purified from 200 g of fresh sponge (Table 1). This lectin could be repeatedly extracted from the precipitate of the sponge after the initial extraction. At low concentrations (<50 μg/mL), the hemagglutination activity increased proportionally with the lectin concentration (Figure 2A and Supplementary Figure S3). hRTL exhibited high thermotolerance (Figure 2B), with thermal stability similar to CCL [10].

The sugar-binding specificity of hRTL is summarized in Table 2. The type-1 β-galactoside, such as the TF-disaccharide (Galβ1-3GalNAc), was the most potent inhibitor of hemagglutination by hRTL (1.6 mM), and the type-2 β-galactoside, such as lactose (Galβ1-4Glc), was also found to be an inhibitor (3.2 mM), though two times less than the former. However, hemagglutination was less inhibited by α-galactoside, such as melibiose (Galα1-6Glc) (50 mM). D-galactose and N-acetyl D-galactosamine moderately inhibited hemagglutination, whereas N-acetyl D-glucosamine and D-mannose did not lead to any inhibition. Porcine stomach mucin and fetuin strongly inhibited the hemagglutination of hRTL, whereas bovine submaxillary mucin was found to be a non-inhibitor.

### 2.2. Characterization of the Primary Sequence of hRTL

Using an automated gas-phase protein sequencer for the Edman degradation, we identified the N-terminal amino acid sequence of hRTL either as DYIEFES (the major sequence) and EYVEVES (the minor sequence) (Supplementary Figure S1). These results indicate that the N-terminus of the lectin was not blocked. The determination of the N-terminal end of the lectin sequence was paired with the analysis of the de novo transcriptome assembly of *C. australiensis*, which we report for the first time in this work, with the aim to identify the transcript encoding the full-length hRTL protein. Overall, RNA sequencing, carried out on an Illumina platform, led to the generation of over 3.0 × 10^7^ high-quality raw paired-end reads (Supplementary Figure S2A,B), which were deposited in a public repository (NCBI BioProject: PRJNA1140828) and assembled to 152,299 unigenes. Overall, both the high quality of the reads and the completeness of the transcriptome assembly (BUSCO scores indicated the presence 92.2% complete, 1.7% fragmented, and 6.1% missing conserved metazoan single-copy orthologs) supported the reliability of this resource as a database for sequence homology searches in this species.

**Figure 3:**
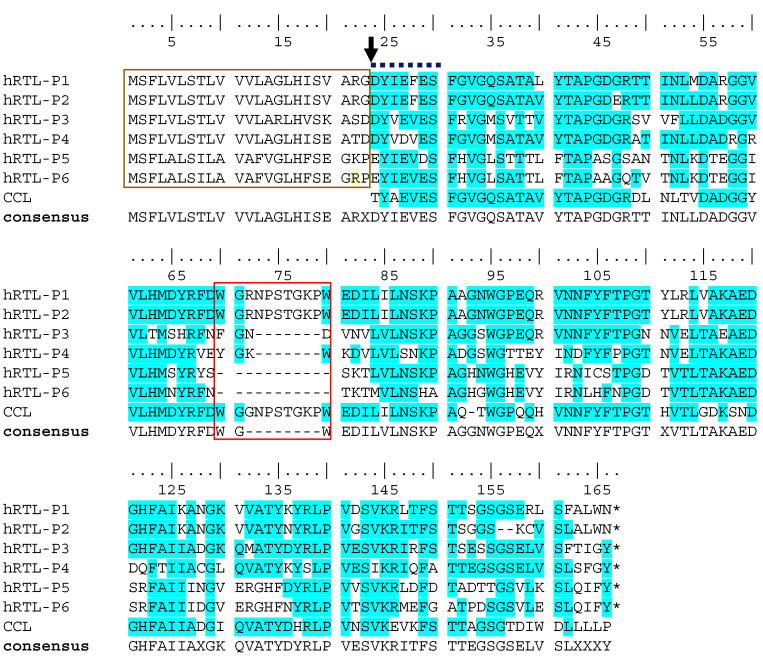
Multiple sequence alignment of the six hRTLs identified in the *C. australiensis* transcriptome. CCL indicates the lectin previously described in *C. calibensis* [10]. Two peculiar sequence features of hRTLs are marked with a gold box (the extension located upstream of the N-terminal end determined by Edman degradation) and a red box (the hRTL-P1-, hRTL-P2-, and CCL-specific insertion). An arrow indicates the position of the N-terminal end of the mature protein determined by Edman degradation. A bold dashed line indicates the partial sequence determined by Edman degradation. Conserved amino acids in the multiple sequence alignment are highlighted with a blue background. The numbering of amino acids starts from the N-terminus at the top. Sequence gaps are indicated by dashes (-). X indicates the presence of any amino acid in the sequence consensus.

A tBLASTn search allowed for the identification of the complete protein sequence of two candidate hRTL precursor sequences, termed hRTL-P1 and hRTL-P2, confirming the correctness of the major sequence determined by the Edman degradation. The inspection of the complete precursors identified the presence of 23 additional amino acid residues located at the N-terminal end, before the N-terminal residue detected by the Edman degradation (Figure 3). In addition, sequence homology searches allowed for the recovery of four additional complete sequences related to hRTL (named hRTL_P3 to hRTL_P6). These sequences had high pairwise primary sequence homology and a similar length (Figure 3), and they were also closely related to the CCL galectin, previously purified from another sponge species belonging to the same genera, *C. caribensis* (Figure 3, CCL). Like hRTL-P1 and hRTL-P2, the other four galectin sequences of *C. australiensis* also displayed 23 amino acids located before the N-terminus determined by the Edman degradation (Figure 3, gold box).

By omitting these 23 amino acids, the calculated molecular masses of hRTL_P1 and P2 (15,526.23 and 15,534.87) would be well-matched with the molecular mass measured by the mass spectrometry of hRTL, i.e., 15,521.11 (Figure 1C), emerging as the best candidates to fit the hRTL lectin sequence purified from sponge extracts. Interestingly, compared with the other four hRTLs, these two proteins also displayed the presence of a 10 amino acid-long insertion, which was shared with CCL (Figure 3, red box).

**Figure 4:**
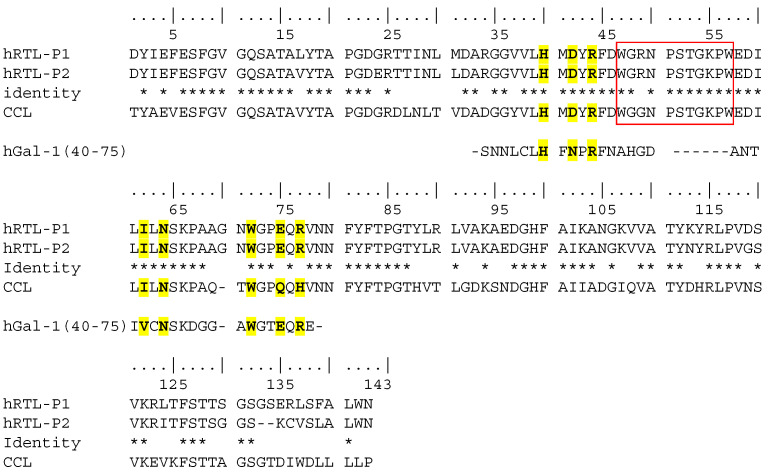
Comparison among the primary sequences of hRTL-P1, P2, and the proto-type *C. caribensis* galectin CCL represents the mature protein sequence only (i.e., after proteolytic cleavage of the signal peptide). Deduced conserved glycan-binding sites are marked in yellow compared to human galectin-1 (hGal-1) [26]. Identical amino acids among hRTL-P1, hRTL-P2 and CCL are indicated by *.

Overall, hRTL-P1, hRTL-P2, and CCL share seven highly conserved amino acids that are important for carbohydrate binding in most galectins, as exemplified by their conservation also in human galectin-1 (Figure 4, yellow highlight). Interestingly, the inserted sequence that distinguishes these lectins from the other hRTLs of *Chondrilla* (Trp47-Trp57) corresponds to an insertion also compared to human galectin-1 (Figure 4, red box).

### 2.3. hRTLs Target the Secretory Pathway by Signal Peptides

**Figure 5:**
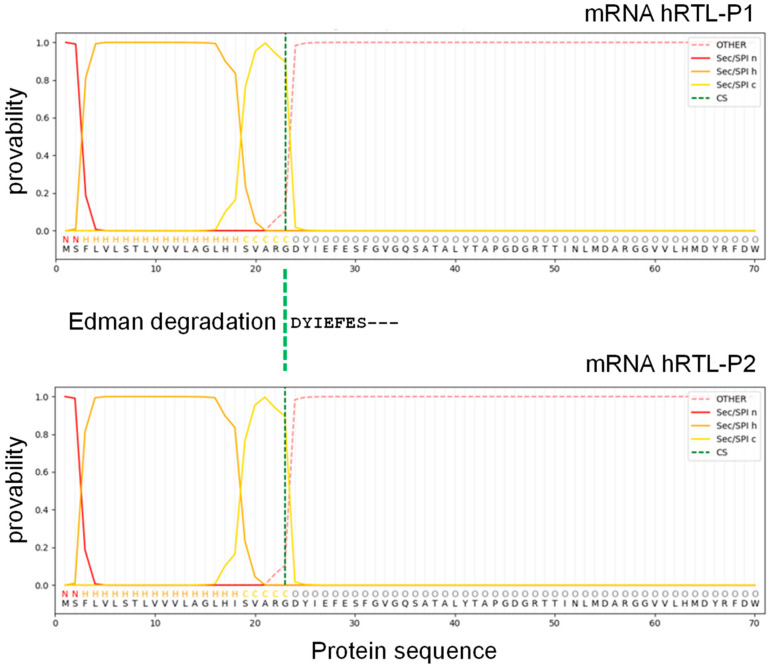
Signal peptide sequence prediction of hRTL. The mRNA sequence was assigned to SignalP 6.0 software. N (red), H (orange), and C (yellow) mean N-region, H-region, and C-region of the signal sequence, respectively.

The SignalP algorithm predicted with high confidence that the 23 amino acid-long extension located at the N-terminal end of the protein sequences of hRTL-P1 and hRTL-P2 were signal peptides used to target the proteins to the secretory pathway and proteolytically cleaved-off during protein maturation. The predicted cleavage site (Gly22) identified by SignalP corresponded precisely to the N-terminal amino acid of the purified mature hRTL (Asp23) detected by the Edman degradation (Figure 5). The other four *C. australiensis* galectins, hRTL-P3 to -P6 sequences, also shared the presence of signal peptides (Supplementary Figure S3).

### 2.4. Glycan-Binding Properties of hRTL

**Figure 6:**
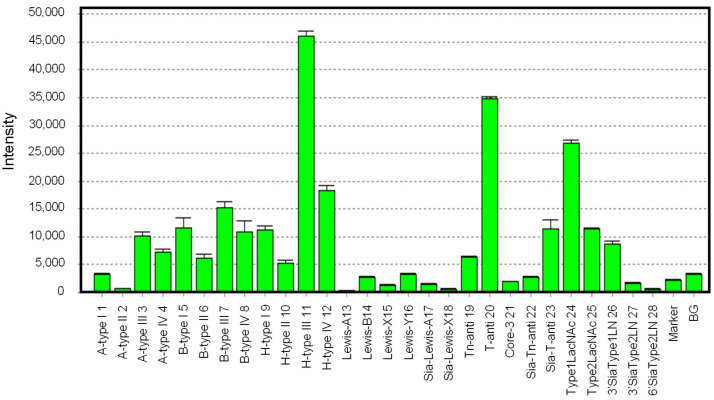
Glycan-binding profile of hRTL. Cy3-labeled hRTL was subjected to glycan array analysis combining a glycan-conjugated array with 28 immobilized glycan structures and a surface plasmon resonance scanning detector (the numbering in the *X*-axis is the same as in Supplementary Table S1). The evanescent-field fluorescence occurring by the binding between Cy3-hRTL and the glycans is represented as a net intensity (*Y*-axis of the graph). BG: background.

The glycan-binding profile of hRTL was determined by glycan array analysis using 28 glycans (Supplementary Table S1), as shown in Figure 6. hRTL showed the highest binding to oligosaccharides of blood type H-glycans, especially those of type-3 (Fucα1-2Galβ1-3GalNAcα1-R) (**11**). The lectin could also bind to those of type-4 (Fucα1-2Galβ1-4GalNAcβ1-) (**12**), type-1 (Fucα1-2Galβ1-3GlcNAcβ1-) (**9**), and type-2 (Fucα1-2Galβ1-4GlcNAcβ1-) (**10**) glycans, but with much lower affinity. However, the link of other monosaccharides to the C-3 position of the non-reducing terminal Gal of type-3 (Galβ1-3GalNAcα1-) glycans, such as in the case of blood type A type-3 (GalNAcα1-3[Fucα1-2]Galβ1-3GalNAcα1-) (**3**) and blood type B type-3 (Galα1-3[Fucα1-2]Galβ1-3GalNAcα1-) (**7**), led to significantly less binding by the lectin.

hRTL displayed a remarkable binding to Thomsen–Friedenreich (TF)-disaccharide (Galβ1-3GalNAc) (**20**), which is known as a cancer-specific glycan associated with mucin. Similar to the case of the type-3 A and B blood group glycans mentioned above, the binding of the lectin to the sialyl-TF-disaccharide (Siacα2-3Galβ1-3GalNAc) (**23**) was highly reduced. Compared to the strong binding to the TF-disaccharide, hRTL was only poorly bound to Tn (GalNAc) (**21**) and sialyl-Tn (Siacα2-3GalNAc) (**22**) antigens, which are also common cancer-specific mucin-associated glycans. This property was consistent with hRTL and was not strongly inhibited by GalNAc compared with Gal (Table 2). Furthermore, the lectin displayed a moderate recognition ability towards type-1 LacNAc (Galβ1-3GlcNAc) (**24**), which was higher than that towards type-2 LacNAc (Galβ1-4GlcNAc) (**25**). This property was also consistent with the stronger binding displayed against type-1 (**1**, **5**, **9**) compared with type-2 (**2**, **6**, **10**) glycans associated with the ABO(H) blood groups (**1**-**12**).

Regarding type-1 LacNAc, the binding of hRTL to Le^a^, where Fuc is bound to the C4 position of GlcNAc (**13**), and Le^b^, where Fuc is bound to the C3 position of Gal (**14**), was significantly reduced, similar to the binding reduction observed for Le^x^ (**15**) and Le^y^ (**16**), which are composed of type-2 LacNAc. This property was consistent with the fact that the binding of hRTL was reduced in sialyl type-1 LacNAc (**26**) compared to type-1 LacNAc. The binding of hRTL to α2-3sialyl type-2 LacNAc (**27**) had an even lower affinity than α2-3 sialyl type-1 LacNAc. The complete abolishment of the binding to α2-6 sialylated Gal (**28**) by hRTL. In summary, among all the tested glycans, hRTL preferentially recognized Galβ1-3GalNAc.

### 2.5. The Cytotoxic Activity of hRTL

**Figure 7:**
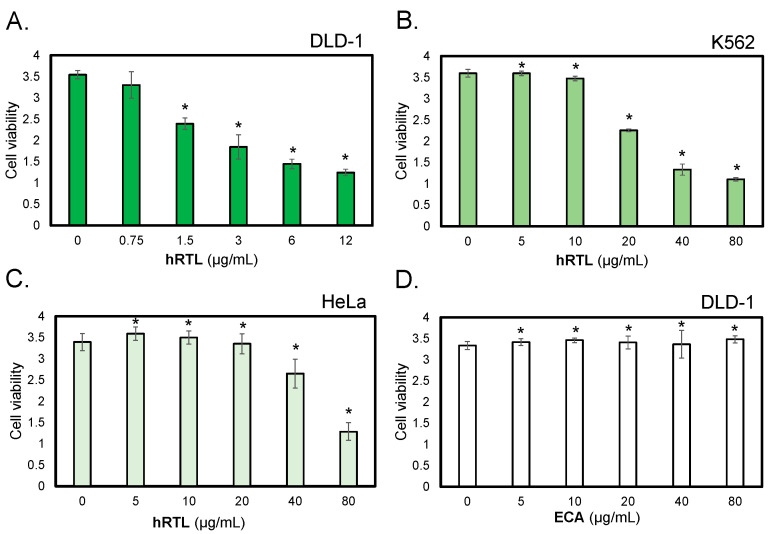
The cytotoxic effects of hRTL against TF-antigen-positive cells. The cytotoxic effects of lectins were evaluated on human colorectal carcinoma cells (DLD-1, panels (**A**,**D**)), human leukemic cells (K562, panel (**B**)), and human ovarian cancer cells (HeLa, panel (**C**)). Cells were treated with hRTL (**A**–**C**) or ECA (**D**); a negative control provided by a lectin that binds to type-1/2 LacNAc at various concentrations (0–80 µg/mL) for 24 h, and cell viability was determined by WST-8 assay. The data shown are the mean ± SE (*n* = 3). *p* values (* *p* < 0.05).

We investigated the cytotoxicity of hRTL against DLD-1, human colorectal carcinoma cells that are known to highly express the TF-antigen [27,28]. In addition, to compare the cellular effect of hRTL, K562 leukemia cells and HeLa cervical cancer cells were selected for the experiments, which express the β-galactoside-containing glycans and moderately express the TF-antigen. Cells (1 × 10^5^/mL) were incubated with various concentrations of hRTL (0.75–80 µg/mL) for 48 h, and cell viability and the proportion of living cells were determined by the WST-8 assay. Cell growth inhibition was observed after adding hRTL at various concentrations, depending on the tested cell line (Figure 7A–C). In particular, the lectin strongly reduced the growth of the colorectal carcinoma DLD-1 cells at low concentrations, leading to a significant effect at 0.75 µg/mL (Figure 7A). On the other hand, another LacNAc-binding lectin, ECA (*Erythrina cristagalli* lectin), which was used in this case as a negative control, did not affect the DLD-1 cells even at much higher concentrations, up to 80 µg/mL (Figure 7D).

**Figure 8:**
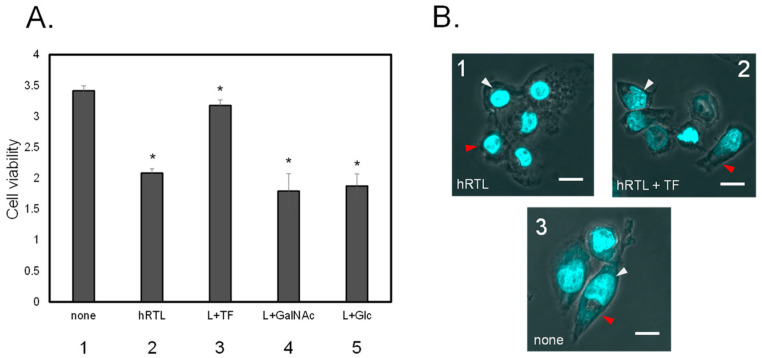
(**A**) Inhibition of cytotoxic effect of 3 µg/mL hRTL on DLD-1. Without (1) or with (2) hRTL (10 µg/mL). Co-presence of TF-antigen, GalNAc, or Glc (each 20 mM: 3–5). All data are reported as mean ± SE (*n* = 3). * *p* < 0.05. (**B**) The morphological appearance of the cells in presence (1 and 2) or absence (3) of the hRTL lectin, the simultaneous presence of 20 mM TF-antigen and hRTL (2). Cell shrinkage and extensions are indicated by red arrows, whereas nucleus condensation is shown with white arrows. Magnification: 40×. Scale bar: 10 µm.

The co-presence of the TF-antigen at 20 mM abrogated the cytotoxicity of hRTL (3 µg/mL) against DLD-1 (Figure 8A, 3), but this effect was not observed in the co-presence of GalNAc or Glc (Figure 8A, 4 or 5). Prior to lectin administration, the shape of DLD-1 cells appeared to be elongated in the culture medium (Figure 8B, 3). However, adding hRTL (3 µg/mL) caused the cells to shrink (Figure 8B, 1). This morphological change was rescued by adding 10 mM of the TF-antigen (Figure 8B, 2).

### 2.6. Prediction of the 3D Structure of hRTLs

**Figure 9:**
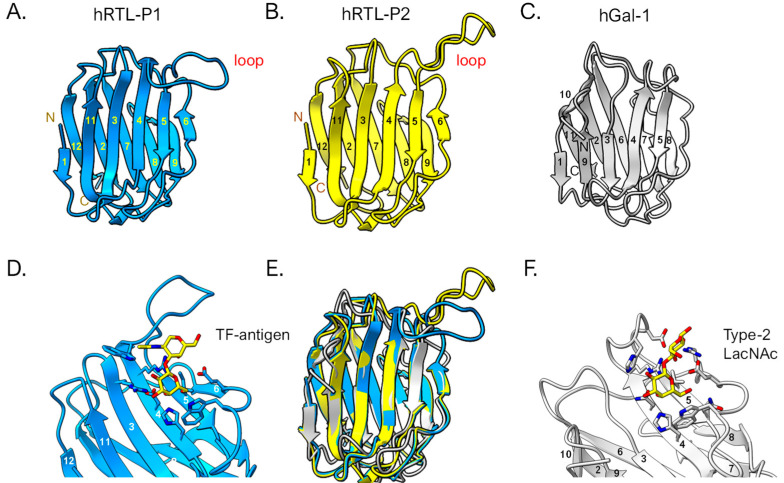
3D structural prediction of hRTLs. hRTL-P1 (**A**) and hRTL-P2 (**B**). hGal-1 (human galectin-1) is also shown as a comparison (**C**). The binding between hRTL-P1 and TF-antigen (**D**), and hGal-1 and LacNAc (**F**), respectively. A superimposed view among hRTL-P1, hRTL-P2, and hGal-1 (**E**). The loops found in the two *Chondrilla* galectins (upper right blue and yellow in panels (**A**,**B**)) were unique characteristics of these hRTLs. Numbers are responding to the order of β-sheets in the polypeptide.

The predicted 3D structures of the hRTL-P1 and P2 were graphically represented with the ChimeraX software version 1.8 [29] (Figure 9). The tertiary structure of these sponge galectins was overall similar to that of human galectin-1 (Figure 9A–C). However, when the structures were superimposed, the hRTLs displayed an accessory loop (Trp47-Trp57), which was not present in hGal-1 (Figure 9E). The loop appeared to cover the binding site of the TF-antigen (Figure 9D). The size of this loop was larger than the loop found in hGal-1, which covered type-2 LacNAc (Figure 9F).

## 3. Discussion

The primary sequence of hRTLs, isolated from the Oceanian sponge *C. australiensis*, displayed several exceptional features that are most likely linked by the proto-typical nature of a galectin family from a basal metazoan phylum. The mature protein sequence of hRTL is a rare example of a galectin with a free N-terminal end, in contrast to most galectins, which typically have this site acetylated. Interestingly, this characteristic is not shared by all sponge galectins, as other lectins in this family isolated from different sponge species have a blocked N-terminus [14]. However, this same feature is shared by CCL, another galectin previously purified from a congeneric species [10], demonstrating multiple occurrences of this unusual phenomenon in sponges.

Combining N-terminal amino acid determination using chemical identification by the Edman degradation with the identification of the full-length protein precursor from transcriptomic data, we were able to detect the presence of six different hRTL sequences sharing high pairwise homology. All six hRTLs had an accessory 23 amino acid-long N-terminal sequence, which was predicted to serve as a signal peptide for secretion with high confidence by SignalP. Furthermore, the signal peptide cleavage site determined by computational prediction matched the N-terminal starting site determined by the Edman degradation (Figure 5), providing a strong confirmation of in silico predictions and supporting the usefulness of the combined multidisciplinary approach we used for the in-depth characterization of hRTL. The presence of a signal peptide is quite an unusual feature within the galectin family, since the overwhelming majority of its members are targeted in the extracellular environment through a non-canonical secretory route, which does not require an N-terminal hydrophobic signal [30]. Only a few exceptions of galectins bearing a signal peptide have been previously reported in Porifera (in a single lectin from the sponge *Geodia cydonium*) and Nematoda (in six and eleven galectins from *Caenorhabditis elegans* and *Strongyloides ratti*, respectively) [17,31,32], highlighting a similarity to the situation we have recently described for mytilectins, another lectin family that may or may not include a signal peptide for secretion in different phyla [33].

The glycan array established that hRTL preferentially recognized type-3 (Galβ1-3GalNAc) and type-1 galactosides, such as the TF-antigen and lacto N-biose, inducing cytotoxicity in gastric cancer cells, such as DLD-1, which expresses the mucin-associated TF-antigen [25]. The solid cytotoxic activity of hRTL might be influenced by its tetrameric configuration, compared to many proto-type galectins, which display dimeric configurations (Figure 1). We plan to bioengineer hRTL to transform it into a monomeric or dimeric form. The comparison between the activity of these different forms will clarify the relationship between cytotoxicity and the arrangement of the lectin in multimeric structures. Proto-type galectins have multifunctional features, such as the ability to induce signal transduction upon binding FGF receptor-1-associated glycans, leading to apoptosis, cell adhesion, and differentiation [34].

Studying the relationships between galectin multimerization and the exerted cell regulative activities will pave the way to biotechnological applications for galectins. Besides the signal peptide, both hRTL-P1 and hRTL-P2 displayed an unusual insertion (Trp45-Trp55), which was not shared by most other functionally characterized galectins. Curiously, this insertion was only present in two of the six hRTLs of *C. australiensis* and CCL. The graphical representation of the 3D structure of the protein allowed for the determination of the structural organization of this region, which assumed to be a loop shape and covered the TF-antigen-binding site of hRTL. We have now begun producing *Chondrilla* galectins using biotechnological procedures for the crystal structure analysis. This will elucidate its high-temperature resistance in the sponge galectin, its binding to the TF-antigen glycans, and its anticancer cell activity mediated by the glycan. The research on galectins has a long history. However, research using sponge galectins to uncover new knowledge about galectins through structural simulations and determination of their 3D has only just started.

## 4. Materials and Methods

### 4.1. Purification of β-D-Galactoside-Binding Lectin from C. australiensis

The marine sponge *Chondrilla australiensis* was collected from the intertidal zone of Sagami Bay, Miura City, Kanagawa Prefecture, Japan. Based on national and local fishing regulations, no permits were required to collect the animals. The fresh sponges were maintained in an aquarium. They were cut into small pieces and homogenized. The purification method of a *Halichondria okadai* lectin, HOL-30, was applied for the purification procedure [16]. A single-edged razor minced two hundred grams (wet weight) of the sponge. Then, it was homogenized with ten volumes (*w*/*v*) of sodium bicarbonate in saline buffer (100 mM sodium bicarbonate containing 150 mM NaCl) containing 10 mM of a protease inhibitor mixture (Fuji film Wako, Osaka, Japan). The homogenates were centrifuged at 14,720× *g* in 500 mL bottles for one hour at 4 °C with a Suprema 21 centrifuge equipped with an NA-18HS rotor (TOMY Co., Ltd., Tokyo, Japan). The supernatant was centrifuged again at 27,500× *g* for one hour at 4 °C, and the clear supernatant was applied to the affinity column of lactosyl-agarose (5 mL) (Sigma-Aldrich, St. Louis, MO, USA). The unbound protein was extensively washed with sodium bicarbonate solution. The lectin was eluted with 50 mM lactose-containing sodium bicarbonate solution, and each 1 mL of elute was collected in tubes with a fraction collector (Model AC-5700, ATTO Co., Ltd., Tokyo, Japan).

The purity of lectin was determined by SDS-PAGE [35]. The eluted fraction was heated at 80 °C for 3 min or 20 min. The aliquots (10 µmL) and the sample buffer (3 µmL) were applied to each well of a mini-slab gel (c-PAGEL: 60 mm × 60 mm with 1 mm thickness; 15% polyacrylamide gel, ATTO Co., Ltd., Tokyo, Japan). After electrophoresis, the gel was treated by CGP staining with 0.1% (*w*/*v*) Coomassie Brilliant Blue (CBB) G-250 in 40% polyvinyl pyrrolidone and 20% citrate acid [36]. Protein concentrations were quantified using a micro-BCA protein assay kit (Thermo Fisher/Pierce, Waltham, MA, USA) based on the principle of bicinchoninic acid for colorimetric detection [37,38], using bovine serum albumin as a standard by measuring A570 (reference: A600) with a microplate reader (model iMark; Bio-Rad Laboratories, Hercules, CA, USA).

### 4.2. Hemagglutinating Activity and Analysis of Sugar-Binding Property

Hemagglutination of lectin was performed in 96-well V-shaped plates as described previously [39]. Initially, 20 µL of two-fold serial dilution of purified lectin in Tris-buffered saline (TBS) was mixed with 20 µL of a 1% suspension (in TBS; *v*/*v*) of trypsinized, glutaraldehyde-fixed rabbit erythrocytes. The TBS and TBS with 0.01% Triton X-100 were used as controls. Plates were kept for 1 h at room temperature. Hemagglutination was observed and scored as a lectin titer.

The saccharide solutions (200 mM, 20 µL) were serially diluted with TBS and 20 µL of each lectin solution was added (adjusted to hemagglutination units 16), along with Triton X-100 (0.01% final conc.) and erythrocytes. Plates were kept for 1 h at room temperature, and minimal inhibitory sugar concentration was determined.

### 4.3. Glycan-Binding Profiling of the Lectin

Glycan array analysis was performed by a system of Glycotechnica Co., Ltd. (Yokohama, Japan). The lectin was fluorescence-labeled (λex/em 560/580 nm) using Cy3 labeling kit-NH_2_ (Cytiva, Tokyo, Japan) per the manufacturer’s instructions. A total of 28 glycans were immobilized on wells of a microarray. Fluorescence-labeled lectins at concentrations ranging from 0 to 100 µg/mL were incubated overnight at 4 °C in the dark. The glycan-binding specificities of the lectin were detected by a GlycoLyte2200 Model evanescent fluorescence scanner (Glycotechnica Co., Ltd., Yokohama, Japan) by modifications to the method of the previous report [40,41].

### 4.4. Molecular Mass Analysis

The purified lectin (5 µg) was subjected to gel permeation chromatography (GPC) utilizing a Shodex KW 402.5-4F column (4.6 mm × 300 mm) connected to an HPLC pump LC-2000 (JASCO Co., Ltd., Tokyo, Japan). The lectin was mixed in 50 mM sodium bicarbonate in saline, and the elution time of lectins from the column was detected by UV at an absorbance of 220 nm.

Matrix-assisted laser desorption/ionization–time-of-flight mass spectrometry (MALDI–TOF-MS) analysis was performed using an AXIMA confidence instrument (Shimadzu) in reflector mode. The purified lectin was co-crystallized with saturated sinapinic acid containing 0.1% trifluoroacetic acid as a matrix on a 384-well MALDI–MS Sample Plate (TO-476R01). One hundred laser shots measured the MS spectrum [42].

### 4.5. N-Terminal Amino Acid Sequence

The lectin’s N-terminal amino acid sequence was determined using an automated Edman degradation using a protein/peptide sequencer Procise 492HT (Applied Biosystems, Foster City, CA, USA) [43].

### 4.6. RNA Sequencing

The tissue was cut into small pieces (20 mg) using razor blades from the sponge (approximately 10 mm squares) that adhered to the rock. The sample tissue was homogenized in a vial with TRIzol (Thermo Fisher Scientific, Waltham, MA, USA). The total RNA, representing a pool of two individual sponges, was extracted following the manufacturer’s instructions. The quality and quantity of the extracted RNAs were assessed using replicates of 500 ng, and 100 ng of quantified samples was then pooled and subjected to next-generation sequencing (GENEWIZ, Azenta Life Sciences, Shinagawa, Tokyo, Japan) on the Illumina Hiseq System in a 2 × 150 bp paired-end configuration for one lane. The same procedure was performed for 30 ng and 10 ng input samples.

The quality of raw sequencing data was first assessed with FastQC v.0.12.0, which allowed for the setting of the most appropriate trimming parameters for Trimmomatic v.0.40 [44] to remove sequencing adapters, low-quality bases, and failed reads. High-quality trimmed reads were *de novo* assembled using Trinity v.3 [45] with default parameters. Nearly perfect matches with the partial amino acid sequence obtained through the Edman degradation were initially searched by selecting hits with tBLASTn matches, scoring an e-value lower than 0.05. Matching nucleotide sequences were subjected to virtual translation to protein with the Expasy translate tool [46]. The resulting protein sequences were analyzed with SignalP v.6.0 [47] to inspect the presence of a signal peptide. The transcriptome assembly quality was assessed with BUSCO v5.7.1 [48] based on the set of conserved orthologous genes of the Metazoa lineage according to OrthoDB v.10 [49].

### 4.7. Cell Viability and Cytotoxicity Assays

DLD-1, K562, and HeLa cells were maintained in RPMI 1640 supplemented with heat-inactivated FBS 10% (*v*/*v*), penicillin (100 U/mL), and streptomycin (100 μg/mL) at 37 °C. Cytotoxic effects and cell growth following treatment with hRTL at concentrations ranging from 0 to 80 µg/mL were determined using Cell Counting Kit-8 containing WST-8 [50]. Cells (2 × 10^4^, in 90 µL solution) were seeded into 96-well flat-bottom plates and treated with 10 µL lectin for 24 h at 37 °C. To assay the effect on cell growth, to each well was added 10 µL of WST-8 solution, followed by incubation for 4 h at 37 °C. The cell survival rate was determined by measuring A450 (reference: A600) with an iMark microplate reader.

### 4.8. 3D Structure Prediction

The hRTL prediction models were generated using ColabFold [51], a Google Colab-based implementation of AlphaFold2 [52]. The docking of the TF-antigen to hRTL-P1 was performed using RosettaLigand on the Rosetta online server, Rosie [53,54,55,56]. The glycan docking position was selected based on the structures of known galectins, such as hGal-1. The output files were validated by comparing them with known structures. The resulting predicted structures were visualized using ChimeraX ver 1.8 [29].

### 4.9. Statistical Analysis

Experiments were performed in triplicate, and results were presented as mean ± standard error (SE). Data were subjected to a one-way analysis of variance (ANOVA) followed by Dunnett’s test using the SPSS Statistics software package, v. 10 (www.ibm.com/products/spss-statistics, accessed on 29 August 2024). Differences with *p* < 0.05 were considered significant.

## Figures and Tables

**Table 1:** Purification of hRTL from *Chondrilla australiensis*.

Fraction	Titer (HU)	Volume (mL)	Total Activity ^a^	Protein Conc. (mg mL^−1^)	Protein Amount (mg)	Specific Activity ^b^	Purification Ratio (Fold) ^c^	Recovery of Activity (%) ^d^
Crude extract	65,536	500	32,768,000	9.5	4750	13.79	1	100
Purified lectin	262,144	80	20,971,520	0.86	68.8	3810.23	276	64

^a^ Total activity is shown by titer × volume. ^b^ Specific activity is shown by titer/mg of protein. ^c^ Purification ratio is shown by comparing the specific activity value of the crude extract vs. purified lectin. ^d^ Recovery of activity is revealed by comparing total activity value of the crude extract vs. purified lectin.

**Table 2:** Saccharides and glycoprotein specificity of hRTLT ^a^.

Saccharides	Minimum Inhibitory Concentration (mM)
TF-antigen	1.6
Lactose	3.2
Melibiose	50
Sucrose	N.I. ^b^
D-galactose	25
D-GalNAc	>50
D-GlcNAc	N.I.
D-mannose	N.I.
Glycoproteins	Minimum inhibitory concentration (mg/mL)
Fetuin	0.125
Porcine stomach mucin	0.125
Bovine submaxillary mucin	N.I. ^c^

^a^ Titer of hRTL was previously diluted to 16. ^b^ Inhibition did not occur even at 200 mM. ^c^ Bovine submaxillary mucin did not inhibit even at 2 mg/mL.

## Data Availability

All hRTL protein sequences are available in Figure S1.

## Supplementary Materials

The following supporting information can be downloaded at: https://www.mdpi.com/article/10.3390/md22090400/s1, Figure S1: Raw data of primary structure analysis of hRTL by Edman degradation.; Figure S2: Plots and graphical representation of FASTQC per base sequence quality scores; Figure S3: Signal peptide sequence prediction of hRTL-P3 to P6; Table S1: Twenty-eight glycan structures on the glycan array.
