# Supplementary material for: Novel Galectins Purified from the Sponge Chondrilla australiensis: Unique Structural Features and Cytotoxic Effects on Colorectal Cancer Cells Mediated by TF-Antigen Binding
Source: Mar Drugs. 2024 Aug 31;22(9):400. doi: 10.3390/md22090400 (PMC11433124; doi:10.3390/md22090400)
Supplement: Supplementary file 1 [file marinedrugs-22-00400-s001.zip › marinedrugs-3179387-supplementary.pdf]

Suppl. Fig S1 Hayashi et al.

```
                      1234567
hRTL-P1   -ARG DYIEFESFGVGQSATAL-
     P2   -ARG DYIEFESFGVGQSATAV-
     P3   -ASD DYVEVESFRVGMSVTTV-
     P4   -ATD DYVDVESFGVGMSATAV-
     P5   -GKP EYIEVDSFHVGLSTTTL-
     P6   -GRP EYIEVESFHVGLSATTL-

           21   25     30   35   40
```

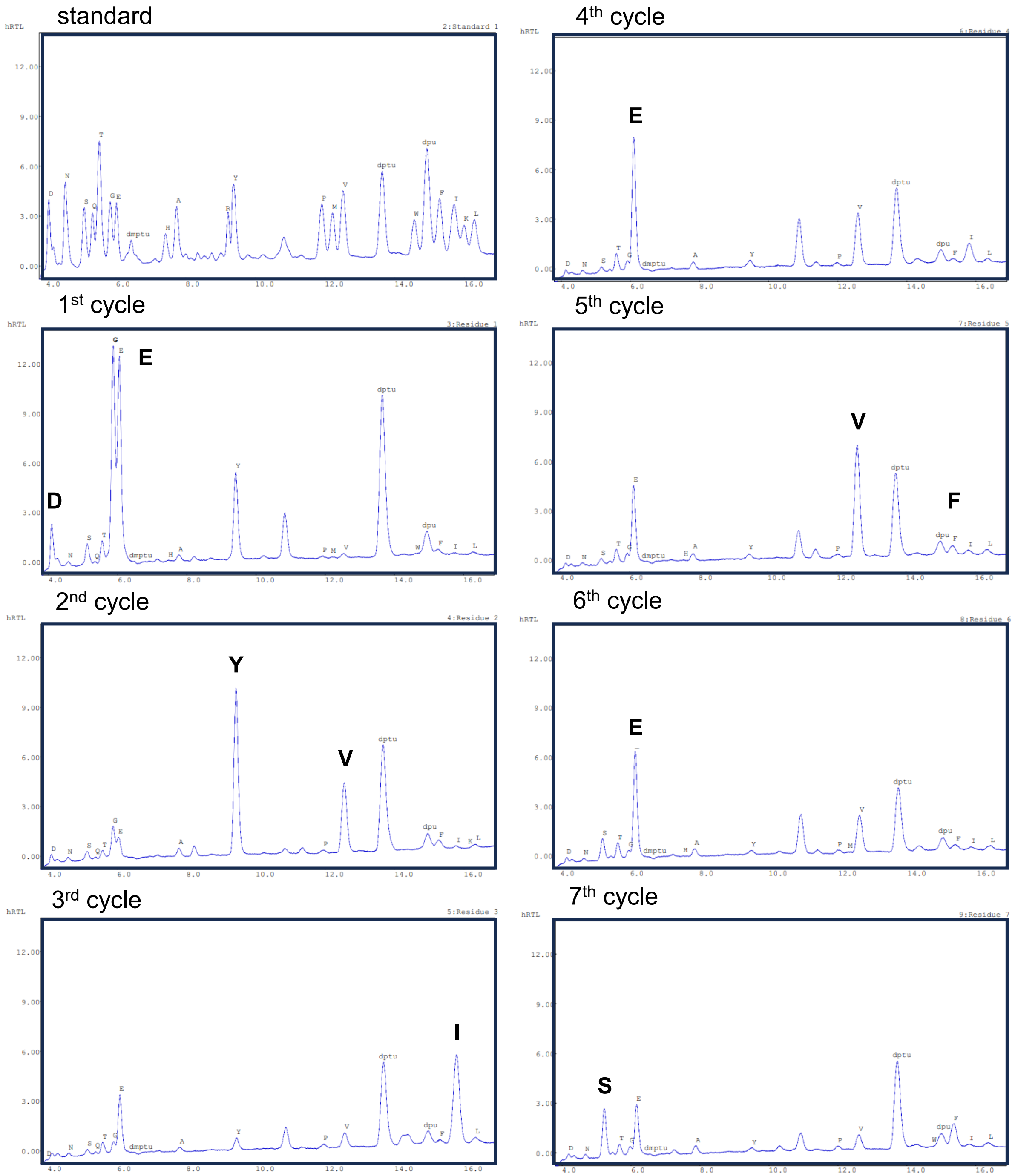

Raw data of primary structure analysis of hRTL by Edman degradation. The top shows Sequences of hRTL-P1 to P6 derived from the transcriptome. Blue numbers indicate the position of N-terminal amino acids in transcripts. Black numbers indicate the cycles of sequencing. Gray indicates amino acids detected by the Edman degradation. Arrow indicates the cleaving site. Chromatograms show raw data of hRTL N-terminal sequencing. Standard shows the elution pattern of PTH-amino acids. Cycles 1-7 show detected PTH-amino acids and their names.

A.

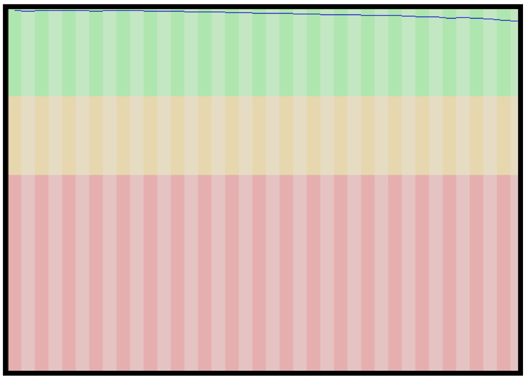

B.

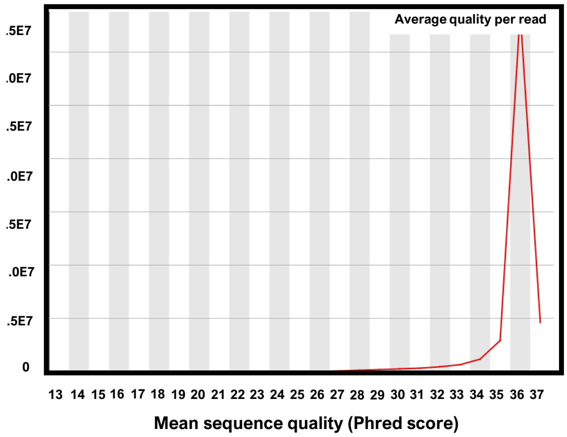

Plots (A) and graphical representation (B) of FASTQC per base sequence quality scores. Data from the RNA sequencing of *C. australiensis*, which was carried out on an Illumina platform and generated over 3.0E7 high-quality raw paired-end reads.

hRTL3

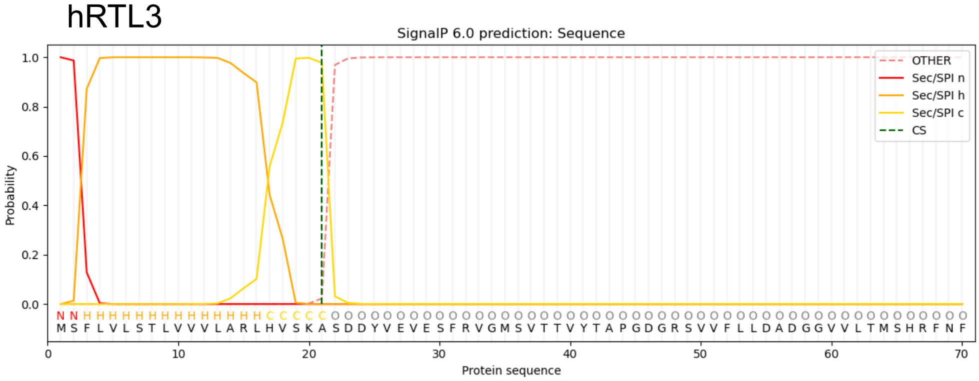

hRTL4

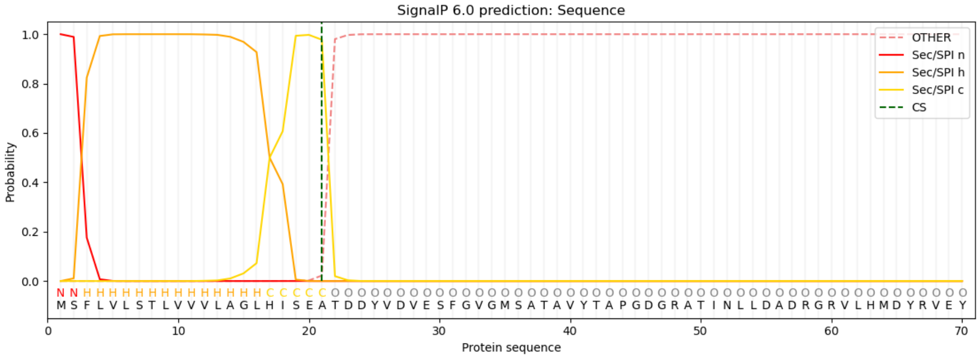

hRTL5

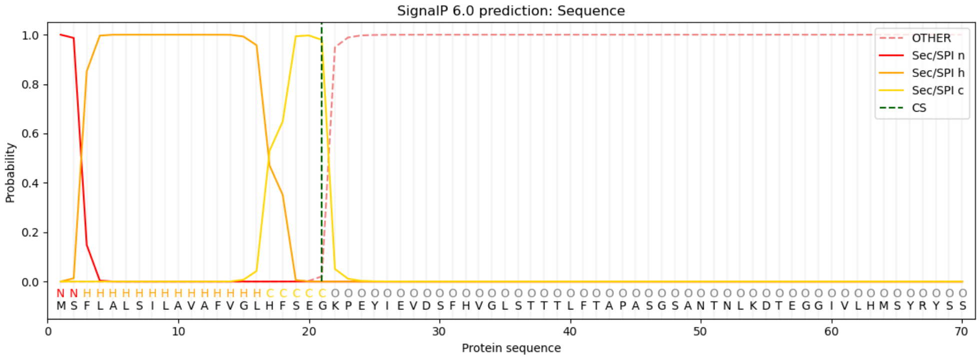

hRTL6

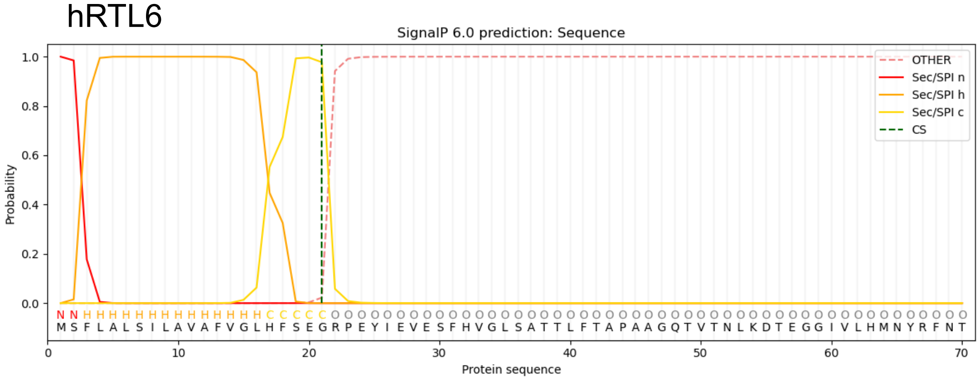

Signal peptide sequence prediction of hRTL-P3 to P6. The mRNA sequence was assigned to SignalP 6.0 software. N (red), H (orange), and C (yellow) mean N-region, H-region, and C-region of the signal sequence, respectively.

Twenty-eight glycan structures on the glycan array.

	Structures	Names		Structures	Names
1	GalNAcα1-3[Fucα1-2]Galβ1-3GlcNAcβ-R[a]	blood A type-1	15	Galβ1-4[Fucα1-4]GlcNAcβ-R	Lewis x
2	GalNAcα1-3[Fucα1-2]Galβ1-4GlcNAcβ-R	blood A type-2	16	Fucα1-2Galβ1-4[Fucα1-3]GlcNAcβ-R	Lewis y
3	GalNAcα1-3[Fucα1-2]Galβ1-3GalNAcα-R	blood A type-3	17	Neu5Acα2-3Galβ1-3[Fucα1-4]GlcNAcβ-R	Sialyl Lewis a
4	GalNAcα1-3[Fucα1-2]Galβ1-4GalNAcβ-R	blood A type-4	18	Neu5Acα2-3Galβ1-4[Fucα1-4]GlcNAcβ-R	Sialyl Lewis x
5	Galα1-3[Fucα1-2]Galβ1-3GlcNAcβ-R	blood B type-1	19	GalNAcα-R	Tn-antigen
6	Galα1-3[Fucα1-2]Galβ1-4GlcNAcβ-R	blood B type-2	20	Galβ1-3GalNAcα-R	TF-antigen
7	Galα1-3[Fucα1-2]Galβ1-3GalNAcα-R	blood B type-3	21	GlcNAcβ1-3GalNAcα-R	mucin core3
8	Galα1-3[Fucα1-2]Galβ1-4GalNAcβ-R	blood B type-4	22	Neu5Acα2-3GalNAcα-R	Sialyl Tn-antigen
9	Fucα1-2Galβ1-3GlcNAcβ-R	blood H type-1	23	Neu5Acα2-3Galβ1-3GalNAcα-R	Sialyl TF-antigen
10	Fucα1-2Galβ1-4GlcNAcβ-R	blood H type-2	24	Galβ1-3GlcNAcβ-R	LacNAc type-1
11	Fucα1-2Galβ1-3GalNAcα-R	blood H type-3	25	Galβ1-4GlcNAcβ-R	LacNAc type-2
12	Fucα1-2Galβ1-4GalNAcβ-R	blood H type-4	26	Neu5Acα2-3Galβ1-3GlcNAcβ-R	3'Sialyl LacNAc type-1
13	Galβ1-3[Fucα1-4]GlcNAcβ-R	Lewis a	27	Neu5Acα2-3Galβ1-4GlcNAcβ-R	3'Sialyl LacNAc type-2
14	Fucα1-2Galβ1-3[Fucα1-4]GlcNAcβ-R	Lewis b	28	Neu5Acα2-6Galβ1-4GlcNAcβ-R	6'Sialyl LacNAc type-2

[a]The reducing terminal residue R is conjugated with 2-amino pyridine.
